# A Meta-Analysis of Randomized Clinical Trials Assessing the Efficacy of PARP Inhibitors in Metastatic Castration-Resistant Prostate Cancer

**DOI:** 10.3390/curroncol30100669

**Published:** 2023-10-19

**Authors:** Zakaria Alameddine, Muhammad Rafay Khan Niazi, Anisha Rajavel, Jai Behgal, Praneeth Reddy Keesari, Ghada Araji, Ahmad Mustafa, Chapman Wei, Abdullah Jahangir, Terenig O Terjanian

**Affiliations:** 1 Staten Island University Hospital, Staten Island, NY 10305, USA; zalameddine@northwell.edu (Z.A.); mniazi@northwell.edu (M.R.K.N.); arajavel@northwell.edu (A.R.); jbehgal@northwell.edu (J.B.); pkeesari@northwell.edu (P.R.K.); garaji@northwell.edu (G.A.); amustafa3@northwell.edu (A.M.); cwei4@northwell.edu (C.W.); 2 University of Oklahoma Health Sciences Center, Oklahoma, OK 73104, USA; abdullah-jahangir@ouhsc.edu

**Keywords:** castrate-resistant prostate cancer, PARP inhibitors, progression-free survival, overall survival, homologous recombination repair genes

## Abstract

Prostate cancer ranks as the second most common malignancy in males. Prostate cancer progressing on androgen deprivation therapy (ADT) is castration-resistant prostate cancer (CRPC). Poly-ADP ribose polymerase (PARP) inhibitors (PARPis) have been at the forefront of the treatment of CRPC. We aim to better characterize the progression-free survival (PFS) and overall survival (OS) in metastatic CRPC patients treated with PARPis. A systemic review search was conducted using National Clinical Trial (NCT), PubMed, Embase, Scopus, and Central Cochrane Registry. The improvement in overall survival was statistically significant, favoring PARPis (hazard ratio (HR) 0.855; 95% confidence interval (CI) 0.752–0.974; *p* = 0.018). The improvement in progression-free survival was also statistically significant, with results favoring PARPis (HR 0.626; 95%CI 0.566–0.692; *p* = 0.000). In a subgroup analysis, similar results were observed where the efficacy of PARPis was evaluated in a subgroup of patients without homologous recombination repair (HRR) gene mutation, which showed improvement in PFS favoring PARPis (HR 0.747; 95%CI 0.0.637–0.877; *p* = 0.000). Our meta-analysis of seven RCTs showed that PARPis significantly increased PFS and OS when used with or without antihormonal agents like abiraterone or enzalutamide.

## 1. Introduction

Prostate cancer ranks as the second most common malignancy in males and is estimated to be responsible for almost 5.5% of all cancer-related deaths in the United States [1,2]. Nevertheless, prognosis and treatment options for advanced disease remain complex [2,3].

Prostate cancer is classically driven by the accumulation of somatic and genetic mutations [1]. This is demonstrated by the higher risk of prostate cancer among the Ashkenazi Jewish population [1,2]. Mutations in genes involved with homologous recombination repair (HRR) are common in advanced prostate cancer, including *BRCA1* and *2* [1,2,4]. Targeting these mutations remains key to effectively treating advanced disease.

The Gleason Score, PSA level, PSA density, percentage of free PSA (free/total PSA ratio (f/t PSA)), and percentage of positive biopsy in core specimens determine the initial staging and prognosis of prostate cancer [2,3,5]. Depending on the life expectancy and symptoms of the patient, initial management for low-risk disease begins with active surveillance for progression [6,7]. The standard of care (SOC) for definitive treatment includes prostatectomy followed by radiation treatment [2,4,8]. Radiation options include external beam radiotherapy (EBRT) or brachytherapy [2]. In higher-risk diseases, radiation is followed by maintenance androgen-deprivation treatment (ADT) using gonadotropin-releasing hormone (GnRH) agonists and antagonists, with agents including leuprolide and goserelin [2,4].

Prostate cancer that progresses on ADT (for example, in the form of increased serum PSA, new metastasis, or progression of pre-existing metastasis) at castrate level testosterone level (<50 ng/dL) is termed castration-resistant prostate cancer (CRPC), and is characterized by changes in androgen receptor (AR) signaling [1,2,4]. CRPC requires additional hormone therapies such as apalutamide and enzalutamide [2,4]. Traditional chemotherapeutic agents, including docetaxel, etoposide, and platinum-based agents, are also used in the setting of metastatic CRPC [2,4,9]. PDL-1 inhibitors and cell therapies such as sipuleucel-T are also available [2,10,11]. In recent years, PARPis have been at the forefront of the treatment of CRPC. PARPis capitalize on mutations in homologous recombination repair (HRR) genes (such as *BRCA1* and *2*, *ATM*), which are frequently found in CRPC [4,12].

PARPis have long been used in patients exhibiting *BRCA1* or *2* mutations in breast and ovarian cancers [13,14,15,16]. PARP1 is a protein that identifies and repairs single-strand breaks (SSBs) in DNA that have been subjected to oxidative stress [15,17,18]. Additionally, PARP1 functions alongside other proteins and via homologous recombination (HR) to repair damaged replication forks and restore DNA replication [17]. Doing so allows cancer cells to continue DNA synthesis and replication. The first PARPi for use in metastatic CRPC was approved by the FDA in 2020 [4].

Several trials have assessed the utility of PARPis in mCRPC [19,20,21,22,23,24,25]. Very recently, a meta-analysis that included Phase II/III studies comprising seventeen trials concluded that the benefit of PARPis was not uniform among the mCRPC patients and showed that the benefit was not uniformly spread between all the patients with alterations in DNA damage repair genes [26]. However, our study included only Phase III trials and showed the benefit of PARPis in improving radiologic PFS in patients with HRR gene alterations and patients lacking it. Previously, a meta-analysis by Niazi et al. that comprised three RCTs revealed significant survival benefits in patients with mCRPC who were treated with PARPis when compared to a placebo or traditional chemotherapies [4]. We performed this meta-analysis to improve the power of the study by Niazi et al., including additional clinical trials that have been performed to date. A subgroup analysis was performed to evaluate the results for patients without HRR gene mutation. The authors hypothesize that PARPis can be extended to a broader cancer population if supported by rigorous prospective trials. We aim to better characterize the progression-free survival (PFS) and overall survival (OS) in mCRPC patients treated with PARPis and determine the subgroup of patients with this disease who can benefit from these medications at maximum.

## 2. Methodology 

The authors of this systematic review followed PRISMA guidelines and adhered to guidelines by the Cochrane Handbook for Systematic Reviews of Interventions in performing this analysis. The study protocol was not registered. 

### 2.1. Search Strategy 

The authors accessed Cochrane Central Registry of Clinical Trials, Embase, Scopus, NCT, and PubMed databases. MeSH (Medical Subject Heading) terms used were PARPi, Prostate cancer, Prostate neoplasm, Olaparib, rucaparib, veliparib, niraparib, talazoparib and docetaxel. The deadline for publication was set as 30 May 2023. 

### 2.2. Inclusion and Exclusion Criteria 

The papers included were as follows:

1. Randomized control trials comparing PARPis with or without androgen receptor pathway inhibitor (ARPI; abiraterone acetate or enzalutamide) against standard of care (ARPI or docetaxel) in prostate cancer patients;
2. Studies that reported progression-free survival and overall survival;
3. Patient age greater than 18 years;
4. Available in the English language without any restrictions on the date or status of the publication.

Papers that did not meet the above criteria were excluded. 

### 2.3. Data Extraction 

Information was extracted using a pre-specified extraction table. Information was filtered from trials through the reading of text and tables. Another author reviewed the information collected to ensure accuracy. The extracted data included Hazard ratios for progression-free survival and overall survival. 

### 2.4. Trial Selection and Evaluation 

Three authors independently reviewed all articles and abstracts and excluded the irrelevant trials. The risk of bias for selected papers was assessed using the Cochrane collaborative tool.

**Figure 1:**
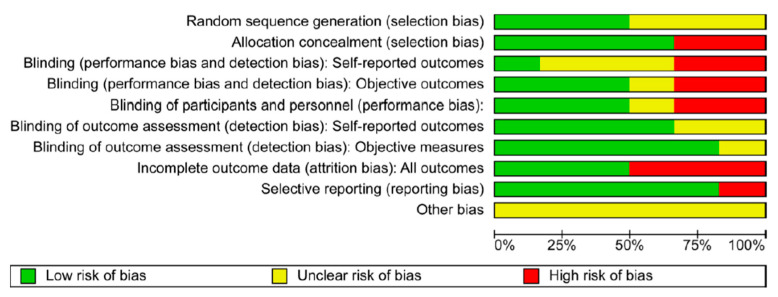
Risks of bias graph: review authors’ judgment about each risk of bias item presented as percentages across all the included studies.

**Figure 2:**
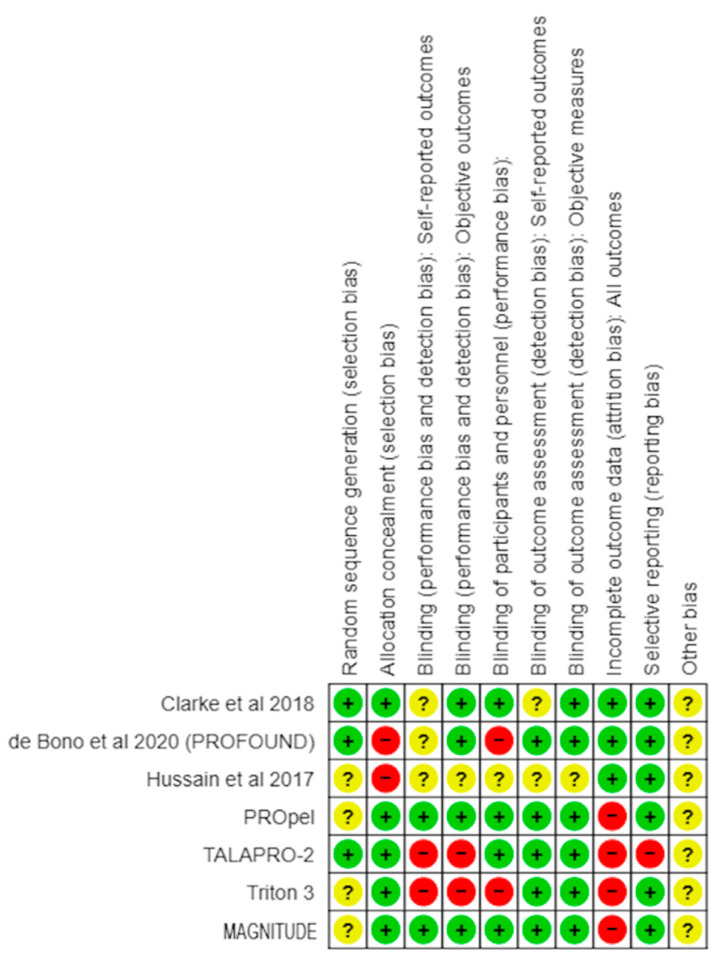
Risk of bias summary: review authors’ judgment about each risk of bias item or each included study. Name of studies are mentioned along vertical axis. Whereas, characteristics are mentioned along horizontal axis. “+” denotes presence of each factor in the study; “−“ denotes absence of that factor” and “?” means that it is uncertain [19,20,21].

Based on this methodology, the risk of biases was classified into high, uncertain, and low (Figure 1 and Figure 2).

### 2.5. Risk of Bias 

Figure 1 and Figure 2 exhibit the risk of bias. 

### 2.6. Study Objectives

The objective of this analysis was to identify all the Phase III randomized controlled trials (RCTs) in which PARPis have been evaluated in the treatment of mCRPC and to compare the efficacy of PARPis among these patients with standard-of-care (SOC)/antihormonal therapies (abiraterone/enzalutamide) or chemotherapy in terms of progression-free survival (PFS) and overall survival (OS). We also aimed to perform an exploratory analysis on the subgroup of these patients who do not harbor HRR gene mutation to investigate the therapeutic efficacy of these agents in terms of PFS in this population.

### 2.7. Statistical Analysis

Comprehensive Meta-Analysis software v. 3 was used to conduct this meta-analysis. Hazard ratios were calculated for PFS and overall survival. For effect sizes, 95%CI (confidence interval) was used, and for statistical significance, a *p*-value of less than 0.05 was used. Heterogeneity was evaluated using I^2^ statistic, with heterogeneity less than 40 considered low, 40–60 considered moderate, and above 60 considered high. Where the median was used, it was assumed to be equivalent to the mean, and SD estimation was obtained by dividing the interquartile difference by 1.35. Fixed-effect analysis is usually adapted in cases where the I^2^ value is ≤50; otherwise, a random-effect model is used. 

## 3. Progression-Free Survival 

### 3.1. Overview 

The analysis was performed on all seven studies. The effect size index was the hazard ratio.

### 3.2. Statistical Model 

Data were analyzed using a random-effect model since the studies were considered an arbitrary sample from a universe of all possible studies. This means that the results of this analysis can be generalized to the larger population of studies.

### 3.3. Mean Effect Size

The mean effect size was 0.630, with a 95% confidence interval of (0.547–0.726). This means we are 95% confident that the true mean effect size falls within this range in the universe of all comparable studies.

The *Z*-test was used to test the null hypothesis that the mean effect size is zero. The *z*-value was −6.415, with a *p*-value of <0.001. As a result, the null hypothesis can be rejected, and it can be concluded that the mean effect size in the universe of populations similar to those in the analysis is not zero.

### 3.4. Q-Test

The Q-statistic is a test of heterogeneity in meta-analysis. It tests the null hypothesis, which means all the studies included in the analysis have a common effect size. If this is true, then the expected value of the Q-statistic is equal to the degrees of freedom (df). In this analysis, the *q*-value was 10.971, with 6 df and *p* = 0.089. This means that the Q-statistic is significantly larger than the expected value, indicating heterogeneity among the studies. Using a criterion α of 0.100, the null hypothesis can be rejected. Hence, we can conclude that the studies do not share a common effect size.

### 3.5. The I^2^ Statistic 

The I^2^ statistic of 45% indicates that 45% of the variation in the observed effects of the studies in the meta-analysis is due to variation in the true effects of the treatment in the different studies, rather than chance (sampling error).

## 4. Overall Survival

### 4.1. Overview 

The analysis was performed on five out of the seven studies. The effect size index was the hazard ratio. 

### 4.2. Statistical Model 

This analysis was performed using the random-effect model. The studies included were considered an arbitrary sample from a universe of potential studies, and this analysis was utilized to make an inference to that universe.

### 4.3. Mean Effect Size

The mean effect size was 0.855, with a 95% confidence interval of (0.752–0.974). This means we are 95% confident that the true mean effect size falls within this range in the universe of all comparable studies. The *Z*-test was used to test the null hypothesis in which the mean effect size would be zero. 

The *z*-value was −2.363, with a *p*-value of less than 0.018. As a result, the null hypothesis can be rejected, and it can be concluded that the mean effect size in the universe of populations similar to those in the analysis is not zero.

### 4.4. Q-Test

The Q-statistic is a test of heterogeneity in meta-analyses. It tests the null hypothesis which means all the studies included in the analysis have a common effect size. If this is true, then the expected value of the Q-statistic is equal to the degrees of freedom (df). The *q*-value was 2.888 with four degrees of freedom.

Since the *q*-value is less than the degrees of freedom, the amount of between-study variance in the observed effects is less than we expect based on sampling error alone. Therefore, true effect variance was estimated as zero, and all heterogeneity indices (I-squared, tau-squared, and tau) were set to zero.

### 4.5. The I^2^ Statistic 

The I^2^ statistic of 0% indicates that 0% of the variation in the observed effects of the studies in the meta-analysis is due to variation in the true effects of the treatment in the different studies, rather than chance (sampling error). 

## 5. Results 

### 5.1. Study Selection and Characteristics 

**Figure 3:**
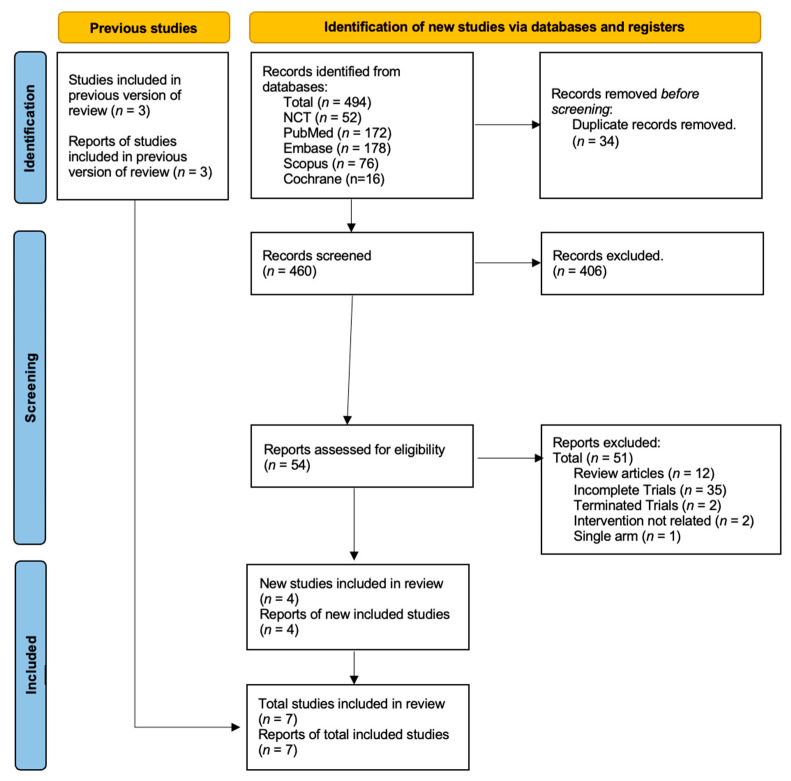
Prisma flow sheet diagram.

After the initial search, 494 articles were identified. Upon removing duplicates, 34 articles were shortlisted, and 406 were filtered out. The full text of 54 articles was analyzed. In total, 35 studies were identified as incomplete trials and hence precluded; 12 were review articles, 2 trials were terminated, 4 were single-arm studies, and 2 studies did not have a relevant intervention. Only seven randomized control trials were included, with 2688 patients. Figure 3 illustrates the PRISMA flow diagram. 

All seven studies used in this analysis were Phase III RCTs. Three of the trials used olaparib, whereas other studies used veliparib, rucaparib, niraparib, and talzoparib in the experimental arm against the standard-of-care treatment. Only four out of these seven studies reported the PFS outcomes for the subgroup of HRR wild-type patients, and that was used in the subgroup analysis. The main features of the included RCTs are listed in Table 1. 

### 5.2. Quality of Studies

Cochrane’s risk of bias tools determined the risk of bias in each study. The “risk of bias graph” shows that the study had a low risk for selection bias secondary to random sequence generation and allocation concealment. Furthermore, studies were double-blinded, decreasing the risk of performance and detection bias. Overall, a low to moderate risk of bias in the studies suggests that the results of this meta-analysis may be subjected to bias. However, the results are still likely to be reliable, as they are based on many studies.

### 5.3. Result of Quantitative Analysis 

#### 5.3.1. Overall Survival (OS) 

**Figure 4:**
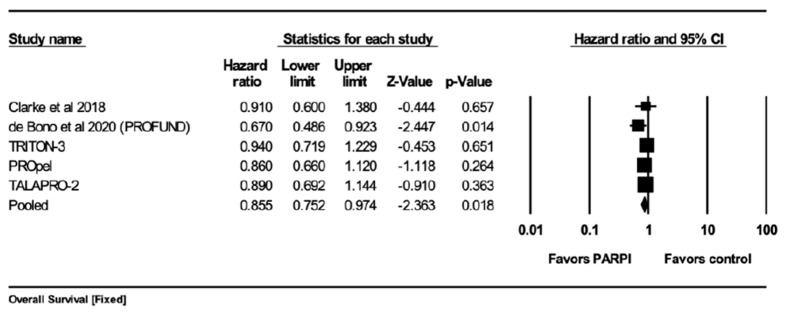
Forrest plot for comparing the overall survival using a fixed-effect model. The mean effect size was 0.855 with a 95% confidence interval of 0.752 to 0.974. The *z*-value was −2.363 with *p* = 0.018. The I-squared statistic was 0% [19,20].

Five studies [19,20,22,23,24] reported overall survival when using PARPis compared with standard of care. The pooled results showed that PFS was significantly better with PARPis (hazard ratio (HR) = 0.855; 95%CI 0.752–0.974; *p* = 0.018) (Figure 4). The pooled analysis was homogeneous (I^2^ = 0%, *q*-value 2.888 with 4 df), and a fixed-effect model was used.

#### 5.3.2. Progression-Free Survival (PFS) 

**Figure 5:**
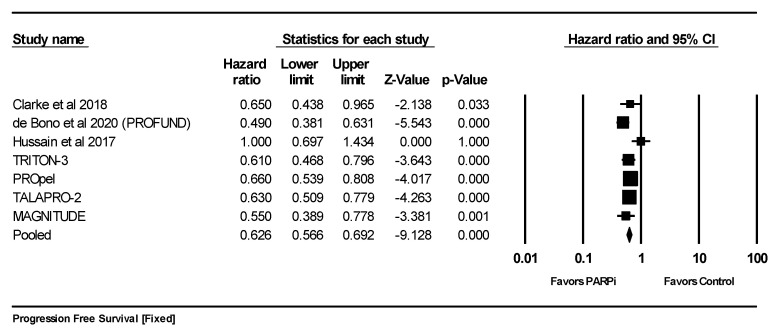
Forrest plot for comparing the progression-free survival using a fixed-effect model. The mean effect size was 0. 626 with a 95% confidence interval between 0.566 and 0.692 with a *z*-value of −9.128 and a *p*-value = 0.000. The improvement in progression-free survival was statistically significant [19,20,21].

Seven studies [19,20,21,22,23,24,25] reported progression-free survival when using PARPis compared with standard of care. The improvement in progression-free survival was found to be statistically significant and favored PARPis (HR 0.626; 95%CI 0.566–0.692; *p* = 0.000). The pooled analysis was homogeneous (I^2^ = 45% and *q*-test for heterogeneity; *p*-value = 0.089), and a fixed-effect model was used (Figure 5).

#### 5.3.3. Progression-Free Survival (PFS) in Patients without HRR Gene Mutation

**Figure 6:**
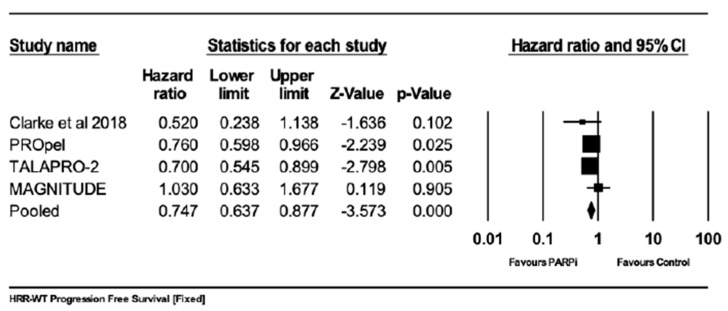
Forrest plot for comparing the progression-free survival in the subgroup of patients without HRR gene mutation. The effect size was 0.747 with a 95% confidence interval between 0.637 and 0.877, with a *z*-value of −3.573, a *p*-value = 0.000, and an I-squared statistics = 0. The results favored PARPis [19].

Four studies reported progression-free survival when using PARPis compared with standard of care in a subgroup of patients without HRR gene mutation. The results favored PARPis (HR 0.747; 95%CI 0.0.637–0.877; *p* = 0.000), and I^2^ = 0 (Figure 6).

## 6. Discussion

Prostate cancer remains the second most common malignancy in males [2]. Current SOC treatment includes prostatectomy, radiation, and ADT [2]. Progression on ADT is termed castration resistance and treatment for metastatic castration-resistant prostate cancer (mCRPC) is less defined [2]. Recently, PARPis have been under investigation to treat mCRPC as monotherapy in second- or third-line treatment options. 

Our meta-analysis of clinical trials revealed that PARPis may improve both PFS (HR 0.626, 95%CI 0.566–0.692, *p* = 0.000) and OS (HR 0.855, 95%CI 0.752–0.974, *p* = 0.018) in patients with mCRPC as compared to placebo or SOC chemotherapy or ADT. These results were computed using the fixed-effect model. The findings also held true using the random-effect model. Moreover, when a subgroup analysis was performed for the patient population who lacked HRR gene mutation, PFS was improved, favoring PARPis (HR 0.747 (0.0.637–0.877), *p* = 0.000) and I^2^ = 0. 

Two PARPis were initially FDA-approved for use in mCRPC in 2020: Rucaparib was approved for use in mCRPC with somatic and/or germline *BRCA1* or *BRCA2* mutations based on TRITON2 [27], and olaparib was approved for mCRPC with HRR gene mutations based on the PROfound trial [20]. So far, as per the NCCN guidelines and FDA approval of rucaparib and olaparib, they are not used as first-line treatment for mCRPC either alone or in combination with ADT. Overall, three trials (BRCAAWAY, MAGNITUDE, and PROPEL) showed improvement in the PFS with PARPis (olaparib, niraparib, and olaparib, respectively) when used in combination with ADT as the first-line treatment for mCRPC, but there was no survival benefit [24,25,28]. Similarly, the results for this combination for patients previously treated with ADT with or without chemotherapy are promising, but it is not recommended outside the clinical trial by NCCN. Numerous other RCTs are currently underway studying the efficacy of PARPis including olaparib, veliparib, rucaparib, niraparib (Zejula), and talazoparib (Talzenna) with and without ADT in mCRPC. Our search of www.clinicaltrials.gov (accessed on 30 May 2023) revealed approximately 39 RCTs studying PARPis in prostate cancer: 19 actively recruiting, 10 completed or nearly completed, and 7 trials with results. 

Three trials investigated the efficacy of olaparib. The first two trials were carried out by Clarke et al. [19] and de Bono et al. [20]. PARPis are effective in mCRPC patients irrespective of the genetic mutation status. However, the analysis performed later by reconstructing the data from the original study revealed that patients in Cohort B (those without HRR genetic alteration) failed to derive any PFS benefit [29]. The study by Clarke et al. aimed to show the benefit of PARPis and novel hormonal agent synergy across all the mCRPC patients regardless of HRR mutation status. Authors hypothesized that the combination of PARPis and novel hormonal agents could be effective in HRR wild-type patients because androgen receptors regulate DNA transcription, and PARP enzyme is used in this process that PARPis can target. Secondly, androgen depletion impairs HRR, producing a BRCA-like phenotype susceptible to PARP inhibition [19]. The third trial is the PROpel study (NCT03732820) [24]. Patients who failed primary androgen deprivation therapy were randomized into those receiving abiraterone and either olaparib or placebo irrespective of HRR mutation status. The study showed significant prolongation in PFS in the treatment group at 24.8 months vs. 16.6 months in the control group (HR 0.66, 95%CI, [0.54–0.81]; *p* < 0.0001). These results exhibit the prospects of PARPis in a broader population of patients with mCRPC, irrespective of HRR mutation status, who have failed earlier line treatment. The overall survival benefit was not significant in this study. Nevertheless, the study results have not matured yet (data maturity: 28.6%). 

The fourth trial investigated the efficacy of veliparib (NCT01576172; Hussain et al.) [21] against the standard of care (abiraterone plus prednisone). The major goal of this trial was to investigate whether ETS fusion status (family of transcription factors) had any effect on tumor response to treatment. Patients were divided into case and control cohorts equally after being classified as having an ETS fusion or not (positive or negative). PFS was found to be similar in both treatment arms. PFS in the treatment group was 11 months (95%CI, 8.1–13.6) vs. 10.1 months (95%CI, 8.2–13.8) in the control group (*p* = 0.99).

The efficacy of rucaparib was originally demonstrated in the Triton-2 trial [27], the results of which were later verified in a Phase III trial (Triton-3) [23]. Patients enrolled in this trial had mCRPC with a *BRCA1*, *BRCA2*, or *ATM* mutation with disease progression after ADT treatment. They were randomly divided into two groups, one receiving rucaparib and the other docetaxel or ADT (abiraterone or enzalutamide). Compared with the previous studies, this was the first study where patients received chemotherapy in the SOC group. The population in the intention-to-treat group comprised patients who had undergone randomization, with a prespecified subgroup of BRCA-mutated patients. There was a significant improvement in the rucaparib group in comparison to the control group in the BRCA analysis and the intention-to-treat (ITT) analysis in BRCA subgroup: 11.2 months (CI, 9.2 to 13.8) vs. 6.4 months (CI, 5.4 to 8.3) (HR, 0.50; 95%CI, 0.36 to 0.69; *p* < 0.001 by log-rank test); in ITT group, patients with the BRCA mutation had greater benefits than those with the ATM mutation. The overall survival benefit was not significant in this study. Nevertheless, the study results have not matured yet (data maturity: 54%). 

Agarwal et al. studied the efficacy of talazoparib, a PARPi that was approved for the treatment of locally advanced or metastatic breast cancer harboring a BRCA mutation, in patients with mCRPC [22]. In TALAPRO-2 (NCT03395197) [22], patients were randomized to receive enzalutamide either with talazoparib or with a placebo. Patients enrolled in this study had disease progression while on androgen deprivation therapy. The results of this study showed a significant improvement in PFS in HRR-deficient (HR, 0.46; 95%CI, 0.30–0.70; *p* < 0.001), HRR-non-deficient or unknown (HR, 0.70; 95%CI, 0.54–0.89; *p* = 0.004), and HRR-non-deficient patients based on tumor tissue testing (HR, 0.66; 95%CI, 0.49–0.91; *p* = 0.009). Hence, this indicates that the addition of talazoparib on enzalutamide had significant improvement in PFS over enzalutamide alone (considered the standard of care) in patients with mCRPC regardless of HRR status. 

The MAGNITUDE trial [25] was a placebo–control study where niraparib was combined with abiraterone and prednisone in the experimental arm and compared with abiraterone/prednisone plus placebo. Patients could have received systemic therapies before enrollment for non-metastatic prostate cancer or metastatic castration-resistant prostate cancer. This study also showed a 45% reduction in rPFS or death. The overall survival data were not immature at the time of publication. In this trial, rPFS was evaluated in the HRR+ cohort with subgroup analysis of *BRCA1*/2-positive patients. A futility analysis for HRR patients was also performed with PARPis, which confirmed no benefit from medication in this population.

Another interesting finding in this study was the subgroup analysis performed on the patients without HRR gene mutation. Out of the seven studies, only four reported the efficacy of the PARPis in terms of PFS in this patient population. The subgroup analysis showed statistically significant improvement in the PFS of this patient population (HR 0.719 (0.607–0.852), *p* = 0.000), an effect that was previously endorsed by the TALAPRO-2 and PROPEL study. These results favor the utility of PARPis in patients with mCRPC regardless of HRR gene mutation status and encourage trials to target a broader patient population who can benefit from the synergy of PARPis and novel antihormonal agents. Authors hypothesized that the combination of PARPis and novel hormonal agents could be effective in HRR wild-type patients because androgen receptors regulate DNA transcription, and PARP enzyme is used in this process that PARPis can target. Hence, the inhibition of the PARP pathway enhances the antiandrogenic effect by suppressing the androgen receptor’s transcription. Secondly, androgen depletion impairs HRR, producing a BRCA-like phenotype susceptible to PARP inhibition [19,30,31].

In contrast to previous studies, the results we observed can translate to meaningful improvement in the overall and progression-free survival in patients with mCRPC. Although the effect was enhanced in the patients who harbored genetic mutations in HRR genes, it still shows a significant improvement in the PFS among patients without HRR gene mutations. In recent RCTs, PARPi was used as a second-line treatment even before chemotherapy and was shown to have promising results. The results of this metanalysis favor the use of PARPis in combination with novel hormonal agents like enzalutamide and abiraterone, regardless of their HRR alterations status, with an acceptable side-effect profile. Additionally, this analysis encourages the consideration of using this combination in upfront treatments for mCRPC rather than in second- or third-line treatment options in which this drug combo can be utilized in a broader population than the patient population currently seen in our clinics who have the potential to derive survival benefits from it.

### Strengths and Limitations

We acknowledge several limitations to this study. These include limited availability of Phase II and III RCTs, as well as the lack of heterogeneity in patient population and narrow inclusionary criteria of patients. Data were derived from only seven studies at the study level, not the patient level. Since the total number of studies was less than 10, publication bias could not be reliably ruled out, which can lead to an overestimation of the results. This can be minimized by including more clinical trials when their results are published. This requires close circumspection while applying the results to the general population. The overall survival data from the newer studies have yet to mature and would require a follow-up of these studies to reanalyze the data. Therefore, the results of this analysis need to be interpreted with caution. Lastly, the study protocol was not registered. Future studies that assess the adverse effects of the medications used in the RCTs would be beneficial. The primary strength of this meta-analysis is that it encompasses all the completed Phase III RCTs.

## 7. Conclusions

Our meta-analysis of clinical trials found that PARPis in combination with novel hormonal agents may improve both progression-free survival (PFS) and overall survival (OS) in patients with metastatic castration-resistant prostate cancer (mCRPC), regardless of their HRR gene mutation status.

These findings suggest that PARPis in combination with novel hormonal agents may be a promising new treatment option for patients with mCRPC, even in patients who do not have HRR gene mutations. This is a significant finding, as it could expand the number of patients who are eligible for PARPi therapy.More research is needed to confirm these findings and to determine the optimal use of PARPis in combination with novel hormonal agents for the treatment of mCRPC.

## Figures and Tables

**Table 1:** Characteristics of randomized control trials (RCTs).

Study Name	Treatment Drugs	Study Characteristic	Inclusion	Exclusion	Primary Outcome
Clarke et al. (NCT01972217) [19]	olaparib (300 mg bid) + abiraterone (1000 mg/od) (*n* = 71) vs. abiraterone (1000 mg/od) alone (*n* = 71)	mCRPC patients previously treated with docetaxel and candidates for abiraterone treatment	Age >18 with mCRPC. ≤2 prior lines of chemotherapy, testosterone <50 ng/dL, no previous exposure to second-generation ARPI, candidates for abiraterone treatment, life expectancy ≥12 weeks, ECOG performance status of 0–2.	Previous treatment with PARPis, or cytotoxic chemotherapy. Other malignancies (including MGUS and MDS) within the last 5 years	Percentage of patients experiencing adverse events Number of patients with dose-limiting toxicities Median (rPFS) time percentage of patients with progression events or death
De Bono et al. PROfound study (NCT029 87543) [20]	olaparib (300 mg bid) vs. enzalutamide (160 mg/od) OR aberaterone (1000 mg/od) + Prednisone (5 mg/bid)	mCRPC patients with disease progression on treatment with enzalutamide or abiraterone Cohort A = Pts with at least one alteration in *BRCA1*, *BRCA2*, or *ATM* Cohort B = Pts with alteration in any of the other 12 genes	men (≥18 years of age) with mCRPC. ≤ 2 prior lines of chemotherapy, no previous exposure to second-generation antihormonal agents, candidates for abiraterone treatment, life expectancy ≥12 weeks, ECOG performance status of 0–2.	Previous treatment with PARPis, or cytotoxic chemotherapy. Other malignancies (including MGUS and MDS) within the last 5 years	PFS via RECIST (v1.1) for soft tissue, as a 20% increase in the sum of diameters of target lesions
Hussain et al. NCT01576172 [21].	Arm A = abiraterone (1000 mg) + prednisone (5 mg/bid) Arm B = veliparib (300 mg/bid)+ abiraterone (1000 mg) + prednisone (5 mg/bid)	pts stratified by ETS fusion status (positive or negative), randomly assigned to Arm (A) and (B)	Men with mCRPC, testosterone <50 ng/dL, ECOG status of 0 to 2, no prior exposure to abiraterone, and up to two prior chemotherapy regimens.	Chemotherapy, radiotherapy, history of active seizures, pituitary or adrenal dysfunction, active or symptomatic viral hepatitis, chronic liver disease, brain metastases	Confirmed PSA response rate time frame: up to 3 years
TRITON-3, NCT02975934	Arm A = oral rucaparib (600 mg twice daily). Arm B = physician’s choice control (docetaxel or a second-generation ARPI (abiraterone acetate or enzalutamide))	Men with mCRPC and a BRCA or ATM alteration + disease progression on previous second-generation ARPI. Previous taxane-based chemotherapy for castration-sensitive disease was permitted.	men (≥18 years of age), with mCRPC, molecular evidence of *BRCA1/2* or *ATM* gene mutation. ECOG 0–1. Disease progression on prior ARPI	Active second malignancy, prior treatment with any PARPi, prior chemotherapy for mCRPC, metastasis to CNS	Assess the efficacy of rucaparib on the basis of rPFS in MCRPC patients with HRD who progressed on prior AR-directed therapy
Propel study NCT03732820	Arm A = oral abiraterone (1000 mg once daily) + olaparib (300 mg twice daily) + prednisone or prednisolone. Arm B = abiraterone + prednisolone + placebo	double-blind, randomized Phase III trial of abiraterone and olaparib versus abiraterone and placebo in first-line treatment of patients with mCRPC regardless of HRR status.	men (≥18 years of age), who are treatment naïve at mCRPC stage, ECOG 0–1, previous treatment with ARPI was allowed if it was at least 4 weeks before randomization	Active second malignancy, MDS or AML, prior treatment with any PARPi.	To determine the efficacy of the combination of olaparib and abiraterone vs. placebo and abiraterone by assessment of rPFS in patients with mCRPC who have received no prior cytotoxic chemotherapy or ARPI at mCRPC stage
TALAPRO-2 (NCT03395197)	Arm A = talazoparib 0.5 mg + enzalutamide 160 mg/daily, Arm B = placebo + enzalutamide 160 mg	pts randomized according to prior abiraterone or docetaxel for CSPC and HRR gene alteration status	Mildly or asymptomatic mCRPC with disease progression at study entry, ECOG PS ≤1, ongoing androgen deprivation therapy, no prior life-prolonging therapy for CRPC	Patients who received treatment at the CRPC stage, prior treatment with PARPis, ARPI, cytotoxic chemotherapy, Brain metastasis	To assess radiologic progression-free survival (rPFS) by BICR per RECIST (v.1.1)
MAGNI-TUDE TRIAL	niraparib 200 mg + abiraterone acetate 1000 mg plus prednisone 10 mg or placebo + AAP	Phase III, randomized, double-blind, placebo-controlled, multicenter study. The efficacy of Niraparib was assessed in HRR+ and HRR-negative patients	Pt who had used ARPI for less than 4 months, prior systemic therapy (docetaxel, enzalutamide, apalutamide, darolutamide) for metastatic castration-sensitive prostate cancer or non-metastatic castration-resistant prostate cancer. No prior use of PARPis	Prior use of PARPis, Use of AAP more than 2–4 months prior to randomization, History of CAD, brain metastasis, or MDS/AML	To evaluate the effectiveness of niraparib and AAP compared to AAP and placebo, as determined by radiographic progression-free survival (rPFS)

ARPI—androgen receptor pathway inhibitor; AR—androgen receptor; HRR—homologous recombination repair; mCRPC—metastatic castration-resistant prostate cancer; ECOG—eastern cooperative oncology group; PARPis—poly(adenosine diphosphate ribose) polymerase inhibitors; MDS—myelodysplastic syndrome; MGUS—monoclonal gammopathy of undetermined significance; rPFS—radiologic progression-free survival; RECIST (v1.1)—response evaluation criteria in solid tumors; PSA—prostate-specific antigen.
